# Thorium and fetal neural tube defects: an epidemiological evidence from large case-control study

**DOI:** 10.1186/s41021-021-00227-w

**Published:** 2021-11-25

**Authors:** Bin Wang, Yiming Pang, Yali Zhang, Le Zhang, Rongwei Ye, Lailai Yan, Zhiwen Li, Aiguo Ren

**Affiliations:** 1 Institute of Reproductive and Child Health, Peking University/ Key Laboratory of Reproductive Health, National Health and Family Planning Commission of the People’s Republic of China, Beijing, 100191 P. R. China; 2 Department of Epidemiology and Biostatistics, School of Public Health, Peking University, Beijing, 100191 P. R. China; 3 Department of Laboratorial Science and Technology, School of Public Health, Peking University, Beijing, 100191 P. R. China

**Keywords:** Neural tube defect, Thorium, Hair, Risk, Coal combustion

## Abstract

**Background:**

Thorium is ubiquitous in the environment and its relationship with birth defects is still under discussion. This study aimed to investigate the associations of maternal exposure to thorium with risk of neural tube defects (NTDs) by using a case–control study, as well as the relationship between thorium exposure and the indoor air pollution from coal combustion.

**Methods:**

This study was conducted in 11 local healthcare hospitals during 2003–2007 in Shanxi and Hebei provinces, China. A total of 774 mothers were included as participants who delivering 263 fetuses with NTDs including 123 with anencephaly, 115 with spina bifida, 18 with encephalocele, and 7 other NTD subtypes (cases), and 511 health fetuses without NTDs (controls). Their hair samples were collected as close as to the occipital posterior scalp, of which those grew from 3 months before to 3 months after conception was cut to measure the thorium concentration by inductively coupled plasma-mass spectrometry.

**Results:**

We found a higher hair thorium concentration in the total NTD cases with 0.901 (0.588–1.382) ng/g hair [median (inter-quartile range)] than that in the controls with a value of 0.621 (0.334–1.058) ng/g hair. Similar results were found for the three concerned NTD subtypes. Maternal hair thorium concentration above its median of the controls was associated with an increased risk of the total NTDs with an adjusted odds ratio of 1.80 [95% confidence interval (CI), 1.23–2.63)] by adjusting for all confounders. There was obvious dose-response relationship between maternal hair thorium concentration and the risk of total NTDs, as well as their two subtypes (i.e. anencephaly and spina bifida). Maternal hair thorium concentration was positive associated with their exposure level to indoor air pollution from coal combustion during cooking.

**Conclusions:**

Overall, our findings revealed that maternal periconceptional thorium exposure was associated with the risk of NTDs in North China. Reducing the coal usage in the household cooking activities may decrease maternal thorium exposure level.

**Graphical abstract:**

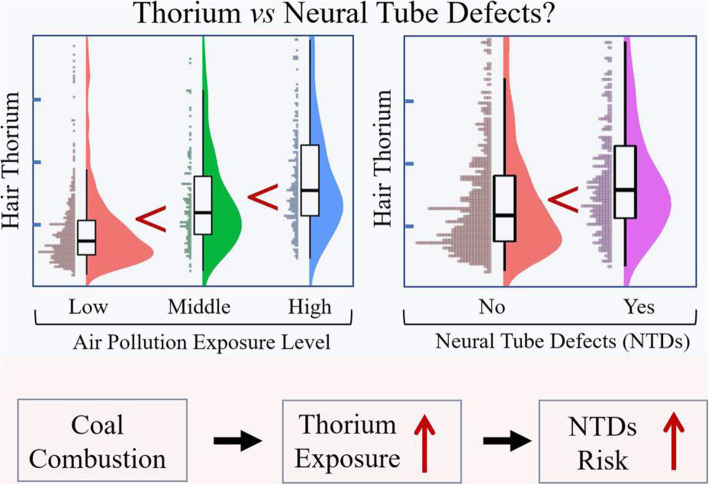

**Supplementary Information:**

The online version contains supplementary material available at 10.1186/s41021-021-00227-w.

## Introduction

Thorium (Th) is a naturally occurring radioactive element and has been used as fuel in nuclear reactors for producing fissionable uranium isotopes. It was estimated that 99% of natural thorium exist in the form of ^232^Th with the half-life of about 14.05 billion years, existing in rocks, soils, water, and sediments, which can cause potential external radiation dose to the public [1–4]. The toxicity of the thorium had been investigated using the in vitro and in vivo models, and its interferences with oxidative stress and immunologic processes were the important toxic pathways [5, 6]. In a general population living without obvious thorium pollution in North China, maternal periconceptional exposure to thorium may be a risk factor for orofacial clefts in offspring [7]. A recent study for the population living near an active U.S. military base in Iraq with high thorium occurrence, there was increased likelihood of congenital anomalies in infants and children, including neural tube defects (NTDs) and congenital heart diseases, associated with their higher thorium exposure [2]. Therefore, the toxic effect of maternal thorium exposure on the birth defects has aroused wide public attention.

NTDs are a group of serious congenital malformations (mainly including anencephaly, spina bifida, and encephalocele) resulting from defective closure of the neural tube by the 28th day post-conception, and the prevalence of NTDs varies by location with the global average of ~ 1/1000 [8]. The prevalence of NTDs in Shanxi and Hebei provinces, located in the North China is the highest in China and worldwide [9–12]. In North China, it was reported that indoor air pollution from coal combustion and the tobacco smoking were both risk factors of fetal NTDs [13, 14]. Though after folic acid supplement, which has been proven to be an efficient way to prevent NTDs occurrence, there were still high prevalence of NTDs in some areas in Shanxi Province, China [12]. It was found that coal mining and combustion is an important exposure route for population to ingest the thorium [15, 16]. In addition, the tobacco smoke also contains certain thorium and has been considered as an non-negligible exposure pathway [17, 18]. Till now, various environmental chemicals from the coal combustion and tobacco smoking had been proposed to be candidate risk factors [19–21]. However, the toxic effect of thorium on NTD development has not been investigated for such population with high NTD prevalence.

Because NTDs mainly form during the very early pregnancy period of ≤4 gestational weeks and the NTD cases were usually confirmed after 12 weeks, it is of high difficulty to collect maternal biological samples (e.g. urine and serum) during the NTD development period to analyze their internal exposure levels of various environmental chemicals. Among them, hair sample has been widely used to indicate population exposure to various environmental pollutants by assuming a relative consistent growth rate [19, 21–25]. For human, various biological samples had been used to represent the exposure level of thorium, including urine, hair, nails, blood, and feces [1, 26, 27]. However, the metabolism pathways were not well addressed. Animal experiment showed that thorium preferentially accumulates in the liver, femur and spleen in mice and exerts its toxic effect at sub-lethal doses [28]. In addition, thorium intake can significantly alter the neurobehaviour of mice and the activity of achetylcholine esterase in the brain with the possible involvement of oxidative damage [29]. It has been proved that hair metals can indicate the population exposure level to thorium in the area with relative high thorium pollution [1, 2]. Therefore, it is possible to analyze the specific hair section close to the NTD development period to characterize maternal thorium exposure. Therefore, the aims of our study are: 1) investigate the relationship between maternal thorium exposure and coal usage during daily life; 2) explore the association between maternal exposure to thorium during the sensitive time window of pregnancy period with the risk of NTDs.

## Materials and method

### Study population

During 2003 to 2007, we conducted a case–control study in four counties including Taigu, Pingding, Xiyang, and Zezhou, as well as Taiyuan City all in Shanxi Province and in six counties including Mancheng, Yuanshi, Shijiazhuang, Laoting, Fengrun, and Xianghe all in Hebei Province. The detailed recruitment method and questionnaire design for the participants have been described previously [25]. Briefly, when a pregnancy (including live births, still births, or pregnancy termination) affected by any NTD subtypes was recruited, one or two women who had delivered a full-term healthy infant without any NTD subtypes in the same hospital were selected as controls. The controls and NTD cases were matched by county/city of residence and date of the last menstrual period. We collected their occipitoposterior hair samples from all participants by cutting the hair as close to the scalp as possible using stainless steel scissor. Each hair sample was sealed in a separate polyethylene zip-lock bag, which was opened prior to the experimental analysis. The related questions from the questionnaire used in this study mainly included maternal age, occupation (“non-chemical industry worker” or “chemical industry worker), education (“primary or lower”, “junior high”, or “high school or above”), gravidity (1, 2, or > 2), history of previous birth defects (“yes” or “no”), periconceptional folic acid supplementation (yes or no), fever or influenza during early pregnancy (“yes” or “no”), alcohol consumption (“yes or “no”), active or passive smoking during the periconceptional period (“yes” or “no”), cooking frequency during the pregnancy period (“frequently” or “rarely”), main fuel for cooking (“coal”, “biomass”, “coal gas”, “liquefied gas”, and “others”,). At last, a total of 774 mothers (511 controls and 263 cases including 123 anencephaly, 115 spina bifida, 18 encephalocele, and 7 other NTD subtype cases) were finally included in this study. The study protocol was approved by the Institutional Review Board at Peking University, and the signed consent was obtained from all participants.

### Hair thorium analysis

By assuming that the rate of hair growth is about 1 cm/month, the hair sections that grew during the period from 3 months before to 3 months after conception (6 months total) were used. The detailed analytical method had been described previously [25]. Briefly, each raw human hair sample (~ 50 mg) was cut into segments 3–5 mm long, and then washed once with 2.5 mL Triton X-100 (by vortexing for 5 min), three times with 2.5 mL deionized water (by vortexing for 5 min), and twice with 3 mL acetone (by vortexing for 5 min). Each hair sample was further digested in a mixture of 0.6 mL nitric acid and 0.4 mL ultrapure water using a microwave accelerated digestion system (MARS 6, CEM Co., USA). Thorium levels were measured using inductively coupled plasma mass spectrometry (ELAN DRC II; Perkin Elmer, USA). The quantification parameters were described in our previous study [30]. The quantitative analysis was conducted by the Central Laboratory of Biological Elements at Peking University Health Science Center in China. The protocol was approved by the China Metrology Accreditation system. The detection limit of hair thorium concentration was 0.03 ng/g hair; the thorium concentrations of all hair samples were all above the detection limit.

### Data analysis

Median value (interquartile range, IQR) was used to describe the hair thorium concentration because it did not follow the normal distribution. Chi-square test was used to compared the characteristics of the case and control mothers, Mann–Whitney *U* test for the differences in thorium concentrations between the cases and controls. NTD status was used as the dependent variable. Hair thorium concentration, used as an independent variable, was dichotomized according to the median value in the control group. To roughly evaluate the indoor air pollution exposure level to the coal combustion due to cooking, the following rules were used to set the air pollution exposure index (EI) based on the questionnaire survey, which was used to roughly distinguish the exposure level to the indoor air pollutants from the coal combustion:

1. If the coal was not the main cooking fuel, EI = 0 when a participant did the cooking frequently or rarely;
2. If the coal was the main cooking fuel, EI = 1 when a participant did the cooking rarely, or EI = 2 when a participant frequently.

This study initially used a matched-pair design to recruit participants, however, the matching was broken due to adding more controls in order to increase statistical power. Therefore, we used an unmatched analysis to estimate the association between maternal thorium exposure and NTD risk. The NTD risk associated with the hair thorium concentration was estimated using odds ratios (ORs) with 95% confidence intervals. An unconditional logistic regression model was used for OR analysis. For the interaction analysis on maternal thorium exposure and folic acid supplementation, maternal hair thorium concentration was dichotomized by its median value in the controls. Thorium concentration quartiles in the controls were used as cutoff values in the dose–response analysis. Statistical analyses were conducted using R software (version 3.6.0, R Development Core Team). A two-tailed *p* value < 0.05 was considered to indicate statistical significance.

## Results

### Population characteristics

The demographic characteristics and hair thorium concentrations of the cases and controls were described in Table 1. There were significant differences between the cases and controls in terms of maternal education background, history of previous birth defects, fever or influenza during early pregnancy, periconceptional folic acid supplementation, passive smoking, cooking frequency, and main fuel type for cooking during the periconceptional period. No differences were found in the other characteristics, including maternal age, occupation, and gravidity. To avoid potential over-matching and to maximize the sample size in the Logistic regression model, the data were analyzed by this study as a loosely matched case–control study. Maternal age, which is commonly viewed as a risk factor for NTDs, along with other characteristics (history of birth defects, education level, occupation, influenza or fever during early pregnancy, folic acid supplementation, and passive smoking during the periconceptional period) in previous studies were included as potential confounders. In consideration that air pollution of coal combustion may be one of the emission sources of thorium, cooking frequency and main fuel type for cooking were not included as confounders, but as the independent risk factor.

**Table 1:** Characteristics and hair thorium concentrations (Conc., unit: ng/g hair) of the recruited mothers who had pregnancies affected by total neural tube defects (Cases),  those who delivered healthy infant (Controls), and the total population (Total)

Group	Controls (*N* = 511) ^a^	Cases (*N* = 263)	Total (*N* = 774)	*P* ^c^	Hair thorium Conc. in Total group (*N* = 774)	*P*
Age (y)						
< 25	233 (45.8) ^b^	118 (45.0)	351 (45.5)	0.510	0.68 (0.38–1.06)	< 0.001 ^d^
25–29	157 (30.8)	75 (28.6)	232 (30.1)		0.56 (0.32–1.01)	
30–34	95 (18.7)	50 (19.1)	145 (18.8)		0.99 (0.61–1.55)	
> 34	24 (4.7)	19 (7.3)	43 (5.6)		1.18 (0.79–1.96)	
Education						
Primary school or lower	39 (7.6)	44 (16.8)	83 (10.7)	< 0.001	1.09 (0.70–1.80)	< 0.001 ^d^
Junior high school	311 (60.9)	176 (67.2)	487 (63.0)		0.81 (0.50–1.26)	
High school	107 (20.9)	34 (13.0)	141 (18.2)		0.40 (0.25–0.69)	
College or higher	54 (10.6)	8 (3.1)	62 (8.0)		0.36 (0.21–0.55)	
Previous birth defects history						
No	501 (98.8)	236 (91.1)	737 (96.2)	< 0.001	0.71 (0.40–1.18)	< 0.001 ^e^
Yes	6 (1.2)	23 (8.9)	29 (3.8)		0.88 (0.57–1.56)	
Occupation						
Non-chemical industry worker	492 (97.2)	225 (94.1)	717 (96.2)	0.062	0.70 (0.40–1.18)	< 0.001 ^e^
Chemical industry worker	14 (2.8)	14 (5.9)	28 (3.8)		0.78 (0.36–1.27)	
Fever or influenza during early pregnancy						
No	487 (97.0)	202 (78.9)	689 (90.9)	< 0.001	0.69 (0.38–1.16)	< 0.001 ^e^
Yes	15 (3.0)	54 (21.1)	69 (9.1)		0.99 (0.66–1.46)	
Periconceptional folic acid supplementation						
No	341 (69.0)	206 (81.1)	547 (73.1)	0.001	0.80 (0.45–1.30)	< 0.001 ^e^
Yes	153 (31.0)	48 (18.9)	201 (26.9)		0.49 (0.26–0.82)	
Passive smoking						
No	354 (69.5)	161 (61.2)	515 (66.7)	0.025	0.70 (0.39–1.16)	< 0.001 ^e^
Yes	155 (30.5)	102 (38.8)	257 (33.3)		0.78 (0.43–1.23)	
Gravidity						
1	224 (44.0)	116 (44.4)	340 (44.2)	0.961	0.66 (0.38–1.05)	< 0.001 ^d^
2	200 (39.3)	100 (38.3)	300 (39.0)		0.78 (0.41–1.23)	
3+	85 (16.7)	45 (17.2)	130 (16.9)		0.89 (0.45–1.42)	
Cooking frequency						
Rarely	256 (53.2)	109 (41.8)	365 (49.2)	0.004	0.59 (0.33–0.96)	< 0.001 ^e^
Frequently	225 (46.8)	152 (58.2)	377 (50.8)		0.88 (0.48–1.42)	
Main fuel type for cooking						
Not coal	303 (59.9)	85 (32.4)	388 (50.5)	< 0.001	0.48 (0.28–0.75)	< 0.001 ^e^
Coal	203 (40.1)	177 (67.6)	380 (49.5)		1.02 (0.69–1.61)	

^a^Number of participants; ^b^ Data are number (percentage). Total number may not be equal to the number of cases or controls due to missing or unknown data; ^c^ Pearson’s *χ*^2^ test, or Fisher's exact test if cell expectation was less than 5.or Fisher's exact test if cell expectation was less than 5; ^d^ Kruskal-Wallis test; ^e^ Mann-Whitney *U* test

### Hair thorium concentration

After stratification analysis by the population characteristics, it showed that there were significant differences of hair thorium concentrations between the subgroups (see Table 1). In the elder participants of ≥30 years old, their hair thorium concentrations were almost two times higher than those of < 30 years old. The participants with low education level also had two times higher of hair thorium concentration than those with relatively high education level. Higher hair thorium concentrations were found in the subgroups with previous birth history, working related to chemical industry, with fever or influenza during early pregnancy, without periconceptional folic acid supplement, more gravidity, and with frequent cooking, and with coal as the main fuel supply for cooking, than those without previous birth history, working without relating to chemical industry, without fever or influenza during early pregnancy, with periconceptional folic acid supplement, fewer gravidity, without frequent cooking, and with other fuel type (not coal) as the main fuel supply for cooking, respectively.

### Association between indoor air pollution exposure and NTD risk

**Fig. 1:**
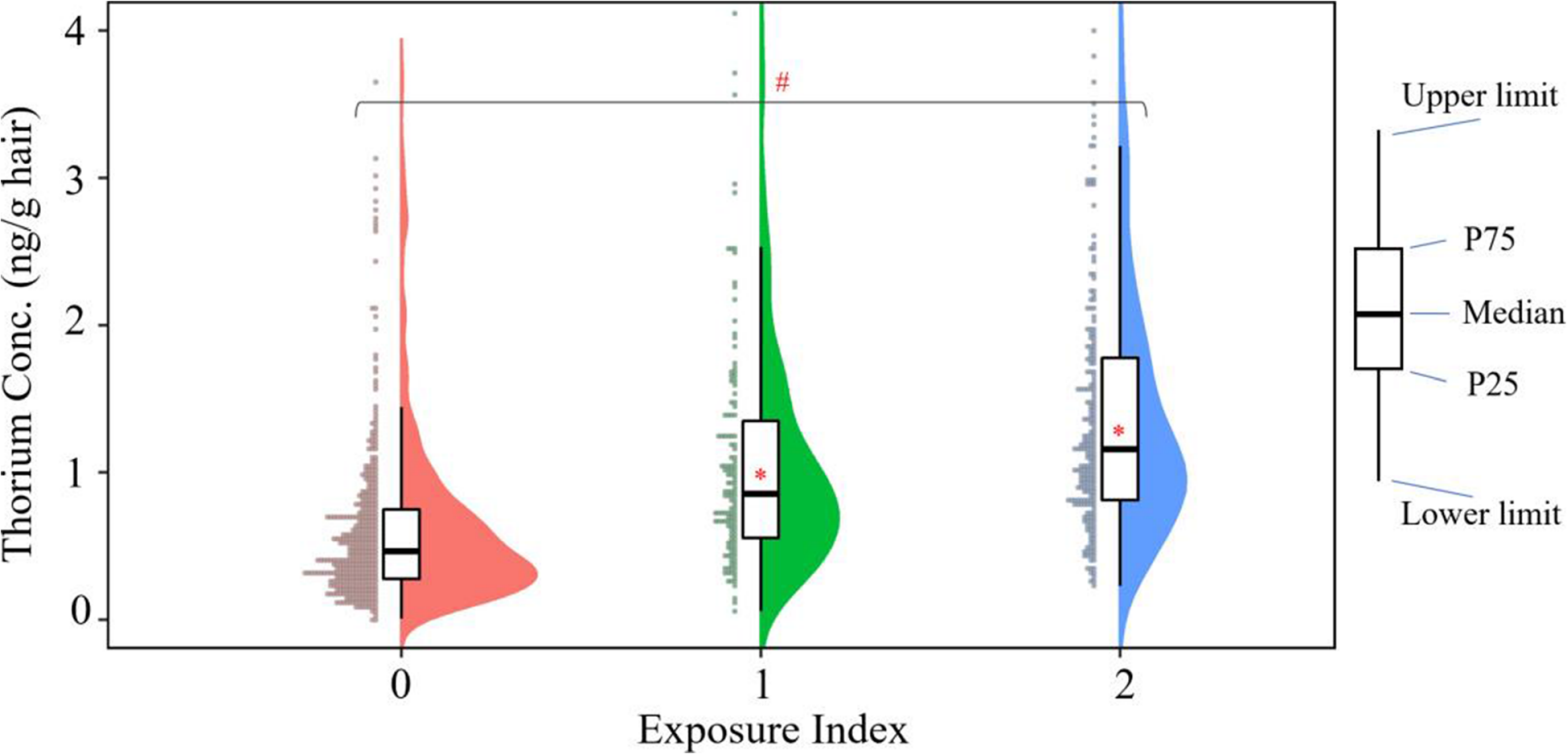
Comparison of hair thorium concentration (Conc.) among the participants with the exposure indices of 0, 1, and 2. The statistical difference between groups were determined by Mann-Whitney *U* test, ^*^ *P* < 0.001. The overall trend was tested by Logistic regression model, ^#^ *P* < 0.001. Data are shown as lower limit value, 25% percentile (P25), median value, 75% percentile (P75), and upper limit value, respectively

In consideration of the potential contribution of the coal combustion pollution, we further compared the hair thorium concentrations among the participants with the EI values of 0, 1, and 2 (see Fig. 1). It showed that there was significant dose-response relationship between hair thorium concentrations and EI. We also found that there was overall dose-response relationship between maternal EI and risks of total NTDs, as well as their two main subtypes, without and with adjusting for confounders (see Table 2).

**Table 2:** Odds ratios of total neural tube defects (NTDs) and two of their subtypes (anencephaly and spina bifida) associated with exposure index (EI) to coal combustion during cooking

EI	Anencephaly (*N* = 123)^a^	P	Spina bifida (*N* = 115)	P	Total NTDs (*N* = 263)	P
	cOR ^b^ (95% CI)					
0	1.00		1.00		1.00	
1	3.60 (2.11–6.15)	< 0.001	3.95 (2.26–6.9)	< 0.001	3.84 (2.53–5.83)	< 0.001
2	3.01 (1.89–4.81)	< 0.001	3.46 (2.13–5.62)	< 0.001	3.18 (2.23–4.55)	< 0.001
	*P*_trend_	< 0.001		< 0.001		< 0.001
	aOR1 ^c^ (95% CI)					
0	1.00		1.00		1.00	
1	1.76 (0.87–3.56)	0.113	2.43 (1.23–4.78)	< 0.05	2.33 (1.40–3.89)	< 0.01
2	1.37 (0.75–2.51)	0.303	2.20 (1.23–3.94)	< 0.01	1.73 (1.11–2.69)	< 0.05
	*P*_trend_	0.270		< 0.01		< 0.01
	aOR2 ^d^ (95% CI)					
0	1.00		1.00		1.00	
1	1.61 (0.78–3.32)	0.199	2.60 (1.14–5.94)	< 0.05	1.95 (1.12–3.39)	< 0.05
2	1.74 (0.83–3.65)	0.143	4.45 (1.98–9.99)	< 0.001	3.10 (1.80–5.36)	< 0.001
	*P*_trend_	< 0.05		< 0.001		< 0.001
	aOR3 ^e^ (95% CI)					
0	1.00		1.00		1.00	
1	1.74 (0.89–3.41)	0.106	2.27 (1.18–4.39)	< 0.05	2.23 (1.37–3.63)	< 0.01
2	1.45 (0.8–2.64)	0.219	2.29 (1.29–4.06)	< 0.01	1.84 (1.19–2.84)	< 0.01
	*P*_trend_	< 0.05		< 0.01		< 0.001

^a^Number of participants; ^b^ Crude odds ratio (cOR) calculated by a logistic regression model; ^c^ Adjusted odds ratio-1 (aOR1) calculated by a logistic regression model adjusted for maternal age, history of previous birth defects, education, occupation, influenza or fever, folic acid supplement, and passive smoking in the periconceptional period; ^d^ aOR2 calculated by a logistic regression model adjusted for maternal age, history of previous birth defects, occupation, influenza or fever, folic acid supplement, and passive smoking in the periconceptional period; ^e^ aOR3 calculated by a logistic regression model adjusted for maternal age, history of previous birth defects, education, occupation, influenza or fever, and passive smoking in the periconceptional period

### Association between hair thorium and NTD risk

**Fig. 2:**
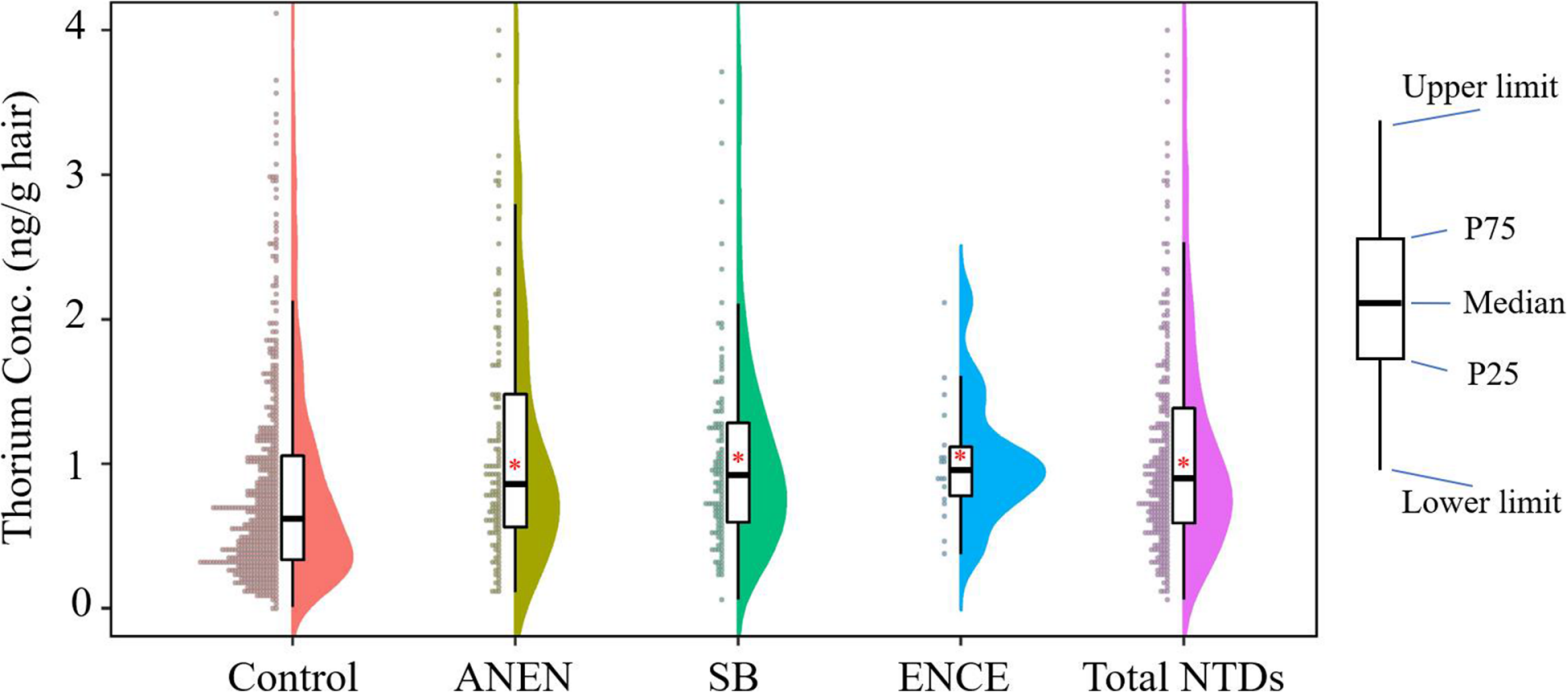
Comparison of hair thorium concentration between the recruited participants who had pregnancies affected by total neural tube defects (Total NTDs), as well as their three subtypes of anencephaly (ANEN), spina bifida (SB), and encephalocele (ENCE), and those who delivered healthy infants (Controls) by Mann-Whitney *U* test, ^*^ *P* < 0.001. Data are shown as lower limit value, 25% percentile (P25), median value, 75% percentile (P75), and upper limit value, respectively

We found a higher hair thorium concentration in the total NTD cases with median (IQR) of 0.901 (0.588–1.382) ng/g hair than that in the controls of 0.621 (0.334–1.058) ng/g hair (see Fig. 2). For the three NTD subtypes, there were also higher hair thorium concentrations, i.e. 0.861 (0.561–1.48) ng/g hair of anencephaly, 0.923 (0.594–1.28) ng/g hair of spina bifida, and 0.956 (0.778–1.12) ng/g hair of encephalocele. As the number of the encephalocele cases is few (i.e. 18 women), their risk assessment associated with hair thorium were not calculated, but they were still included in the total NTD cases.

The calculated ORs for overall NTDs and the two NTD subtypes (anencephaly and spina bifida) associated with hair thorium concentrations are provided in Table 3. The regression analysis indicated that a high hair thorium concentration was a risk factor for NTDs in general (cOR = 2.48, 95% CI: 1.82–3.37), as well as for anencephaly (cOR = 2.19, 95% CI: 1.46–3.28) and spina bifida (cOR = 2.54, 95% CI: 1.67–3.87). Four regression models were adopted by including various confounders (see Table 3). The regression model firstly included the potential confounders of maternal age, history of previous birth defects, education, occupation, influenza or fever, folic acid supplement, and passive smoking in the periconceptional period, and found no significant associations between the hair thorium concentration and anencephaly (aOR1 = 1.40, 95% CI: 0.83–2.35), but spina bifida (aOR1 = 1.96, 95% CI: 1.18–3.24). Due to the linear correlation between education level and occupation (*P* < 0.01 by Spearman correlation analysis), the second regression model excluded maternal education as the confounder. The results were overall consistent with those from the first model. As folic acid supplementation during the periconceptional period were the dominant factor for NTD development, we further excluded it from the confounders and found that folic acid supplementation did not significantly affect the ORs for the overall NTDs or for the two NTD subtypes. After including the exposure index as the confounder, the overall association between hair thorium and risk of total NTDs were consistent.

**Table 3:** Odds ratios of total neural tube defects (NTDs) and two of their subtypes (anencephaly and spina bifida) associated with hair thorium concentrations dichotomized by medians of controls

OR	Anencephaly (*N* = 123) ^a^	*P*	Spina bifida (*N* = 115)	*P*	Total NTDs (*N* = 263)	*P*
cOR ^b^	2.19 (1.46–3.28)	< 0.001	2.54 (1.67–3.87)	< 0.001	2.48 (1.82–3.37)	< 0.001
aOR1 ^c^	1.40 (0.83–2.35)	0.209	1.96 (1.18–3.24)	< 0.01	1.80 (1.23–2.63)	< 0.01
aOR2 ^d^	1.53 (0.93–2.52)	0.097	2.30 (1.41–3.75)	< 0.001	2.08 (1.45–3.00)	< 0.001
aOR3 ^e^	1.53 (0.92–2.55)	0.103	1.95 (1.19–3.19)	< 0.01	1.88 (1.30–2.73)	< 0.001
aOR4 ^f^	1.39 (0.79–2.45)	0.254	1.63 (0.94–2.80)	0.080	1.67 (1.11–2.51)	< 0.05

^a^Number of participants; ^b^ Crude odds ratio (cOR) calculated by a logistic regression model; ^c^ Adjusted odds ratio-1 (aOR1) calculated by a logistic regression model adjusted for maternal age, history of previous birth defects, education, occupation, influenza or fever, folic acid supplement, and passive smoking in the periconceptional period; ^d^ aOR2 calculated by a logistic regression model adjusted for maternal age, history of previous birth defects, occupation, influenza or fever, folic acid supplement, and passive smoking in the periconceptional period; ^e^ aOR3 calculated by a logistic regression model adjusted for maternal age, history of previous birth defects, education, occupation, influenza or fever, and passive smoking in the periconceptional period. ^f^ aOR4 calculated by a logistic regression model adjusted for maternal age, history of previous birth defects, education, occupation, influenza or fever, passive smoking, and exposure index in the periconceptional period

The interaction effect between the hair thorium concentration and folic acid supplementation was not observed using these four regression models (see Table S1). We also classified all participants into four exposure levels based on their quartiles of hair thorium concentration, and calculated their dose-response relationship with risk the total NTDs, as well as those of the two main subtypes (see Table S2). It revealed that there were overall dose-response relationships between hair thorium concentration and the risks of both anencephaly and spina bifida, respectively. For the anencephaly, the significantly toxic effect was found in the highest exposure level, i.e. L4 (> 75th percentile).

## Discussion

Our study investigated the association between maternal thorium exposure and fetal NTD risk by analyzing the thorium concentration in the specific hair sections around the pregnancy grew during the period from 3 months before to 3 months after conception (6 months total). We found that hair thorium concentration of hair was positively associated with the risk of NTDs, as well as their subtypes. The exposure to indoor air pollution from coal combustion during cooking may be an important source of maternal thorium exposure. Our study overall supported that maternal thorium exposure may be an important risk factor to the fetal NTDs development in North China.

Previous studies about hair thorium analysis are limited. However, there were two important case studies to indicate that hair thorium can be used as efficiency exposure biomarker. In Niska Banja, a town located in southern Serbia, with locally high natural background radiation, hair thorium level were found particularly high in the people who lived in the area with the strongest radiation pollution of thorium [1]. Another study found that there was an inverse association between distance to the area of Tallil Air Base and hair thorium level, which was conducted near a US military base around Nasiriyah in Iraq, where war contamination of dispensed bombs, bullets, detonation of chemical and conventional weapons, and burn-pit emissions resulting in high radiation pollution [2]. These evidences from the pollution areas supported us to investigate the association of the maternal exposure to thorium with NTD risk. Overall, the hair thorium concentration of 0.712 (0.397–1.18) ng/g hair [median (IQR)] or 1.28 ± 3.11 (mean ± standard deviation) of the 774 participants in our study were overall lower than those of the serious pollution area of Nasiriyah in Iraq [i.e. 3.73 ± 3.00 ng/g hair (controls) and 6.09 ± 3.22 ng/g hair (cases) (mean ± standard deviation)] [2], but it is comparable with those living in the radiation polluted areas of southern Serbia [i.e. 1.4 (0.16–28) ng/g hair, median (min–max)] [1]. However, the hair thorium concentration in our study were overall in the reference ranges compared in USA (0.2–5.8 ng/g hair) [31] and Sweden (0.3–4.4 ng/g hair) [32]. Overall, the participants in our study were living in the areas without strong thorium pollution.

For the participants in our study, there were two possible exposure pathways of thorium, i.e., passive smoking and indoor air pollution from coal combustion (especially during cooking). Thorium is a primordial radioactive element which is ubiquitous in the environment mediums. It can be released into the atmosphere from both natural and anthropogenic sources, including Earth’s crust, cosmic rays, volcano eruption, nuclear fuel plants, coal combustion, and ore mining and refining [3]. It had been reported that tobacco smoking is one of the important sources of thorium intake into human body [17, 18]. This route is usually overlooked in the epidemiological study. For the potential contribution of coal combustion to the thorium exposure, thorium emission and its risk management had been discussed in China [16] and other countries [4, 15]. It suggested that pregnant women should reduce the exposure change to tobacco smoking and coal combustion environment to lower the thorium exposure level. The adverse effects of indoor air pollution from coal combustion and passive smoking on the NTD risk had been reported in our previous study [13, 14]. This further revealed that the thorium intake from the coal combustion may be one of the important toxic components.

The biological toxicity of thorium had been investigated using various in vitro and in vivo studies. For example, it was found that low concentration of ^232^Th can induce proliferation of human-derived liver cells, which was inhibited by the pre-treatment of cells with neutralizing antibody against insulin-like growth factor 1 receptor [5]. Chronic thorium exposure can cause alterations in the oxidative parameters of silver catfish gills, which are correlated with the thorium accumulation in this organ [6]. However, the related reports about the reproductive toxicity induced by thorium is very rare. Also, there were no reports about the relationship between maternal thorium exposure and risk of NTDs to the best of our knowledge. As for other birth defects, there were two recent published studies. It was reported that living near an active U.S. military base in Iraq is associated with significantly higher hair thorium and increased likelihood of congenital anomalies in infants and children [2]. In North China, maternal periconceptional exposure to thorium may be a risk factor for orofacial clefts in offspring [7]. Our study firstly proposed that maternal periconceptional exposure to thorium may contribute to the development of NTDs. We found that there were no interaction effects between maternal hair thorium concentration and folic acid supplement among these participants. Also, to the best of knowledge, we did not search the related animal study to investigate whether thorium intake can cause the formation of the NTD like phenotype. However, the related study can provide the suggestive information to conduct the confirmation study. It showed that a small fraction (∼3%) of total injected thorium was found to accumulate in the brain of mice, which can cause their neurobehavioural alteration and impairment of cholinergic function through the oxidative stress induction in different brain regions [29]. Our previous epidemiological study revealed that higher oxidative stress level was present in women carrying pregnancies affected by NTDs [33]. The in-depth mechanism should be further investigated.

Our study had three important limitations to be addressed. First, we only analyzed the total thorium content in hair samples, of which the isotopes of thorium (i.e. ^228^Th, ^230^Th, and ^232^Th) were not distinguished. Second, the relationship between population intake level of thorium and its hair concentration was not confirmed by the animal study. Though higher hair Th concentration had been found in the area with severe Th pollution in some areas than those without obvious Th pollution, we consider that it still needs confirming that high Th exposure can cause an increased hair Th concentration using an animal model. Also, it is a relatively appropriate way to explore the potential pathogenic mechanism, which was not also confirmed in our study. Third, we did not distinguish the contributions of Th from other toxic pollutants in the air pollution generated from the coal combustion, some other environmental epidemiological methods should be considered in the future to increase the causal inference, e.g. exposomics [34], repeated measurement analysis [35], and Bayesian model [36]. However, our study had three important advantages. First, the hair sample grown during or close to the period of neural tube closure was analyzed to represent the maternal exposure level to thorium. Second, the sample size of 774 participants including 263 NTD cases and 511 controls was the largest population for the human biomonitoring study to the best of our knowledge to explore the potential role of thorium. Third, our study was conducted in the areas with high NTD prevalence in China. The related results can be directly referred for the local policy-makers to protect the health of the pregnancy women from the NTDs.

## Conclusions

We concluded that maternal periconceptional thorium exposure may contribute to the risk of NTDs in North China. Our study firstly proposed this hypothesis as far as we know. As this finding was indicated in the areas with high NTD prevalence using a large case-control study, we consider that our study warrants further confirmation, especially in areas with a relatively high thorium pollution level.

## Supplementary Information

**Additional file 1: Tables S1-S2**

## Data Availability

The data are available in the main text or the supplementary materials can be obtained by contacting the corresponding author (Dr. Zhiwen Li).

## Abbreviations

NTDs: Neural tube defects
OR: Odds ratio
cOR: Crude odds ratio
aOR: Adjusted odds ratio
CI: Confidence interval
Th: Thorium
EI: Air pollution exposure index
IQR: Interquartile range
