# Supplementary material for: Thorium and fetal neural tube defects: an epidemiological evidence from large case-control study
Source: Genes Environ. 2021 Nov 25;43:51. doi: 10.1186/s41021-021-00227-w (PMC8614024; doi:10.1186/s41021-021-00227-w)
Supplement: Supplementary file 1 — Additional file 1: Tables S1-S2 [file 41021_2021_227_MOESM1_ESM.doc]

**Supplementary Materials**

**Thorium and Fetal Neural Tube Defects: An Epidemiological Evidence from Large Case-control Study**

Bin Wang ^1,2^, Yiming Pang^1,2^, Yali Zhang^1,2^, Le Zhang^1,2^, Rongwei Ye ^1,2^, Lailai Yan ^3,*^, Zhiwen Li ^1,2,*^, Aiguo Ren ^1,2^

^1^ Institute of Reproductive and Child Health, Peking University/ Key Laboratory of Reproductive Health, National Health and Family Planning Commission of the People’s Republic of China, Beijing 100191, P.R. China

^2^ Department of Epidemiology and Biostatistics, School of Public Health, Peking University, Beijing 100191, P. R. China

^3^ Department of Laboratorial Science and Technology, School of Public Health, Peking University, Beijing 100191, P. R. China

^*^ Corresponding Author:

Dr. Lailai Yan, E-mail: yll@bjmu.edu.cn

Dr. Zhiwen Li, E-mail: [lizw@bjmu.edu.cn](mailto:lizw@bjmu.edu.cn)

| Number of tables: | 2 |
| --- | --- |

**Table S1**. Interaction effects between hair thorium (Th) concentration and folic acid supplement status on the risk of neural tube defects (NTDs) and their two main subtypes (anencephaly and spina bifida)

| Variables | Crude OR ^c^  (95% CI) | *P* |  | Adjusted OR ^d^  (95% CI) | *P* |
| --- | --- | --- | --- | --- | --- |
|  | Anencephaly |  |  |  |  |
| Hair Th ^a^ | 1.66 (1.04–2.66) | <0.05 |  | 1.44 (0.82–2.54) | 0.208 |
| Folic acid supplement | 0.48 (0.24–0.97) | <0.05 |  | 0.69 (0.31–1.52) | 0.354 |
| Hair Th × Folic acid supplement ^b^ | 1.75 (0.62–4.93) | 0.288 |  | 0.98 (0.75–1.27) | 0.855 |
|  | Spina bifida |  |  |  |  |
| Hair Th | 2.01 (1.24–3.26) | <0.01 |  | 1.70 (0.98–2.97) | 0.061 |
| Folic acid supplement | 0.42 (0.19–0.92) | <0.05 |  | 0.52 (0.20–1.32) | 0.168 |
| Hair Th × Folic acid supplement | 2.33 (0.80–6.72) | 0.119 |  | 0.88 (0.68–1.15) | 0.356 |
|  | Total NTDs |  |  |  |  |
| Hair Th | 1.89 (1.32–2.70) | <0.01 |  | 1.65 (1.08–2.5) | <0.05 |
| Folic acid supplement | 0.44 (0.26–0.76) | <0.01 |  | 0.60 (0.32–1.11) | 0.106 |
| Hair Th × Folic acid supplement | 2.19 (1.01–4.74) | <0.05 |  | 0.92 (0.75–1.12) | 0.382 |

^a^ Hair Th concentration was classified by the median values, i.e. < 50% percentile (<0.0834 µg/g hair) and ≥ 50th percentile (0.0834 µg/g hair); ^b^ Multiplication interaction term between hair Th and folic acid supplement; ^c^ Calculated by a logistic regression model; ^d^ Calculated by a logistic regression model adjusted for maternal age, history of previous birth defects, education, occupation, influenza or fever, and passive smoking during the periconceptional period.

**Table S2**. Dose-response relationship between hair thorium concentrations and risk of neural tube defects (NTDs), as well as their two main subtypes (i.e. anencephaly and spina bifida)

| Levels | Anencephaly | P | Ptrend | Spina bifida | P | Ptrend | Total NTDs | P | Ptrend |
| --- | --- | --- | --- | --- | --- | --- | --- | --- | --- |
|  | cOR ^b^ (95% CI) | | | | | | | | |
| L1 | 1.00 |  | <0.001 | 1.00 |  | <0.001 | 1.00 |  | <0.001 |
| L2 | 1.95(1.04-3.67) | 0.038 |  | 2.51(1.25-5.06) | <0.01 |  | 2.20(1.36-3.57) | <0.01 |  |
| L3 | 3.01(1.62-5.57) | <0.001 |  | 4.28(2.17-8.43) | <0.001 |  | 3.92(2.45-6.27) | <0.001 |  |
| L4 | 3.25(1.77-5.97) | <0.001 |  | 4.28(2.18-8.40) | <0.001 |  | 3.73(2.33-5.96) | <0.001 |  |
|  | aOR1 ^c^ (95% CI) | | | | | | | | |
| L1 | 1.00 |  | 0.111 | 1.00 |  | <0.05 | 1.00 |  | <0.01 |
| L2 | 1.51(0.73-3.16) | 0.269 |  | 2.14(0.92-4.96) | 0.076 |  | 1.71(0.97-3.00) | 0.061 |  |
| L3 | 1.59(0.74-3.41) | 0.234 |  | 3.44(1.50-7.88) | <0.01 |  | 2.57(1.46-4.51) | <0.001 |  |
| L4 | 1.94(0.92-4.10) | 0.081 |  | 2.96(1.28-6.82) | <0.05 |  | 2.39(1.35-4.22) | <0.01 |  |
|  | aOR2 ^d^ (95% CI) | | | | | | | | |
| L1 | 1.00 |  | <0.05 | 1.00 |  | <0.05 | 1.00 |  | <0.001 |
| L2 | 1.61(0.78-3.32) | 0.199 |  | 2.60(1.14-5.94) | <0.05 |  | 1.95(1.12-3.39) | <0.05 |  |
| L3 | 1.74(0.83-3.65) | 0.143 |  | 4.45(1.98-9.99) | <0.001 |  | 3.10(1.80-5.36) | <0.001 |  |
| L4 | 2.18(1.07-4.43) | 0.032 |  | 3.85(1.70-8.72) | <0.01 |  | 2.98(1.72-5.15) | <0.001 |  |
|  | aOR3 ^e^ (95% CI) | | | | | | | | |
| L1 | 1.00 |  | <0.05 | 1.00 |  | <0.01 | 1.00 |  | <0.001 |
| L2 | 1.54(0.74-3.19) | 0.247 |  | 2.31(1.00-5.29) | <0.05 |  | 1.78(1.02-3.10) | <0.05 |  |
| L3 | 1.74(0.82-3.67) | 0.147 |  | 3.48(1.53-7.91) | <0.01 |  | 2.65(1.52-4.61) | <0.001 |  |
| L4 | 2.16(1.04-4.49) | 0.040 |  | 3.20(1.40-7.29) | <0.01 |  | 2.66(1.52-4.64) | <0.001 |  |
|  | aOR4 ^f^ (95% CI) | | | | | | | | |
| L1 | 1.00 |  |  | 1.00 |  |  | 1.00 |  |  |
| L2 | 1.49(0.71-3.14) | 0.291 | 0.261 | 1.99(0.84-4.67) | 0.116 | 0.115 | 1.64(0.93-2.91) | 0.088 | <0.05 |
| L3 | 1.63(0.74-3.62) | 0.226 |  | 2.92(1.23-6.92) | 0.015 |  | 2.41(1.34-4.34) | 0.003 |  |
| L4 | 1.9(0.86-4.21) | 0.115 |  | 2.27(0.94-5.52) | 0.070 |  | 2.14(1.16-3.93) | 0.014 |  |

^a^ Hair Th concentration was classified by the quartiles of all the 774 participants, i.e. < 25% percentile (< 0.3973ng/g hair), 25th – 50th percentile (0.3973 ~ 0.7121 ng/g hair), 50th – 75th percentile (0.7121 ~ 1.1769 ng/g hair), > 75th percentile (0.150 ng/g hair); ^b^ Crude odds ratio (cOR) calculated by a logistic regression model; ^c^ Adjusted odds ratio-1 (aOR1) calculated by a logistic regression model adjusted for maternal age, history of previous birth defects, education, occupation, influenza or fever, folic acid acid supplement, and passive smoking in the periconceptional period; ^d^ aOR2 calculated by a logistic regression model adjusted for maternal age, history of previous birth defects, occupation, influenza or fever, folic acid supplement, and passive smoking in the periconceptional period; ^e^ aOR3 calculated by a logistic regression model adjusted for maternal age, history of previous birth defects, education, occupation, influenza or fever, and passive smoking in the periconceptional period; ^f^ aOR4 calculated by a logistic regression model adjusted for maternal age, history of previous birth defects, education, occupation, influenza or fever, passive smoking, and exposure index in the periconceptional period.
